# Supplementary material for: Local enrichment of HP1alpha at telomeres alters their structure and regulation of telomere protection
Source: Nat Commun. 2018 Sep 4;9:3583. doi: 10.1038/s41467-018-05840-y (PMC6123478; doi:10.1038/s41467-018-05840-y)
Supplement: Supplementary file 1 — Supplementary Information [file 41467_2018_5840_MOESM1_ESM.pdf]

SUPPLEMENTARY INFORMATION

Local Enrichment of HP1alpha at Telomeres Alters Their Structure and Regulation of Telomere Protection

Chow et al.

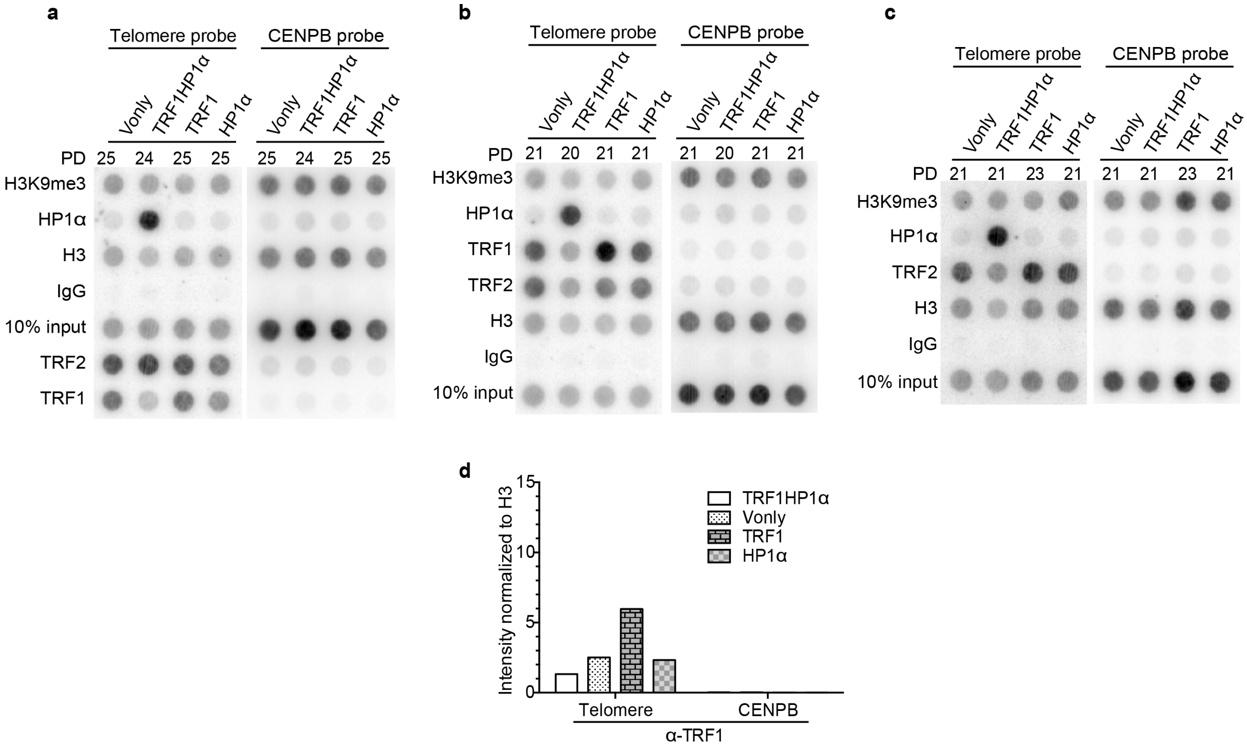

Supplementary Figure 1. Independent replicates of ChIP. (a-c) Uncropped dot blots of experimental samples immunoprecipitated with indicated antibodies, and hybridized with either telomere probes or control centromere (CENPB) probes (*n* = 3). **(a)** The identical blot is shown in Fig. 1f. For the purpose of the flow of the text, the order of the antibodies is rearranged differently in Fig 1f. **(b-c)** independent replicates. **(d)** Upon normalization to H3, TRF1 alone shows an average of ~2.5 fold increase TRF1 at telomeres in comparison to Vonly or HP1α (*n* = 2). The decreased TRF1 signal at the telomeres of TRF1HP1α could be due to limited antibody accessibility due to the particular protein configurations of TRF1HP1α. Thus, TRF1 signal at the telomeres of TRF1HP1α cannot not be evaluated.

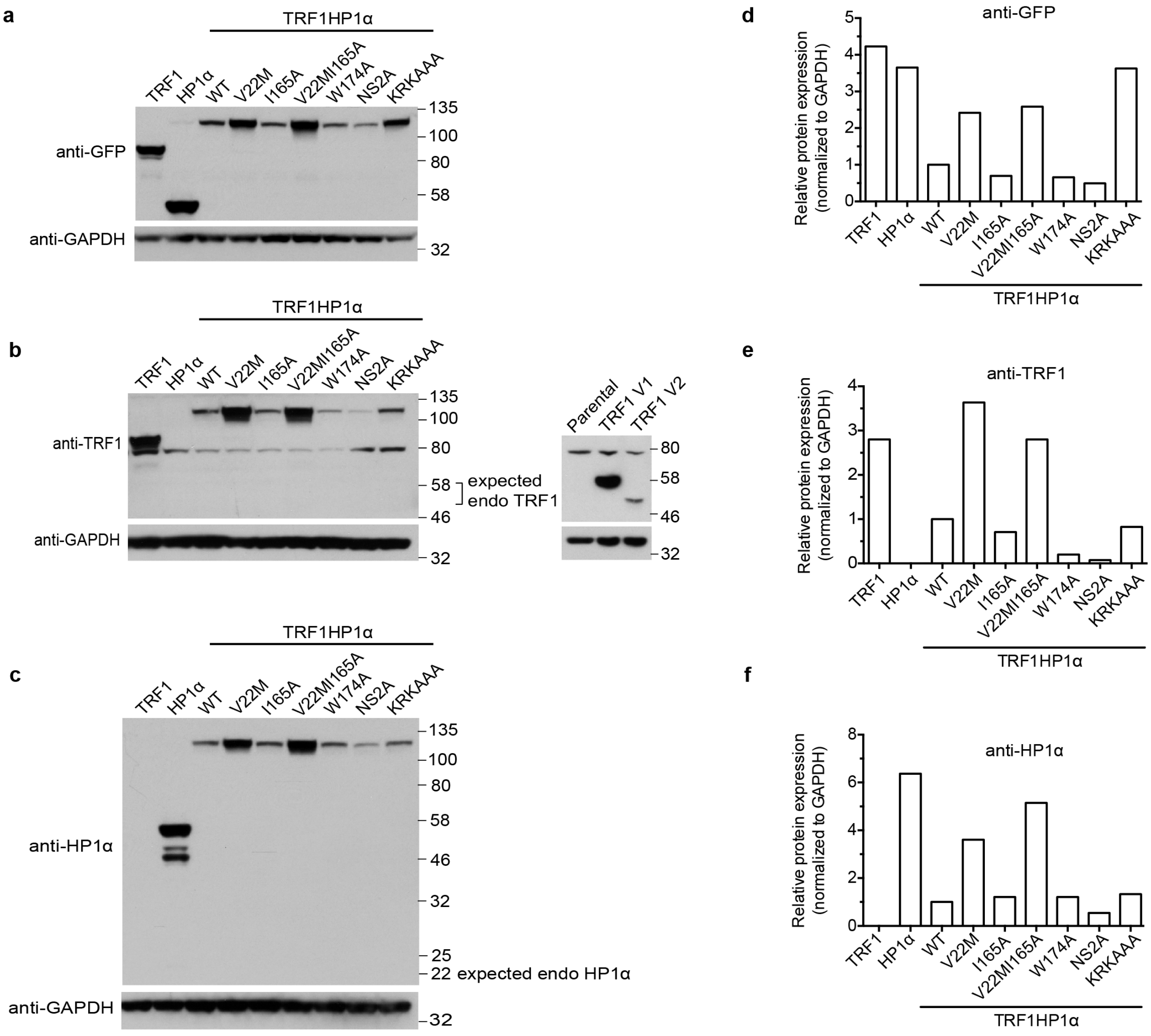

Supplementary Figure 2: Protein expression. Protein overexpression in UM-UC3 cells, measured with **(a)** antibody against GFP (anti-GFP) **(b)** left: antibody against TRF1 (anti-TRF1); right: Specificity validation of TRF1 antibody after transient overexpression of TRF1 variant 1 (V1) NM_017489 and variant 2 (V2) NM_003218 (OriGene Technologies, Inc). Note that the antibody detected both transcript variants when overexpressed, but not endogenous TRF1 (indicated as endo TRF1). **(c)** Antibody against HP1α (anti-HP1α). Anti-GAPDH is used as loading control for **(a-c)**. **(d-f)** Corresponding quantification of protein expression relative to WT TRF1HP1α. Inputs are normalized to GAPDH.

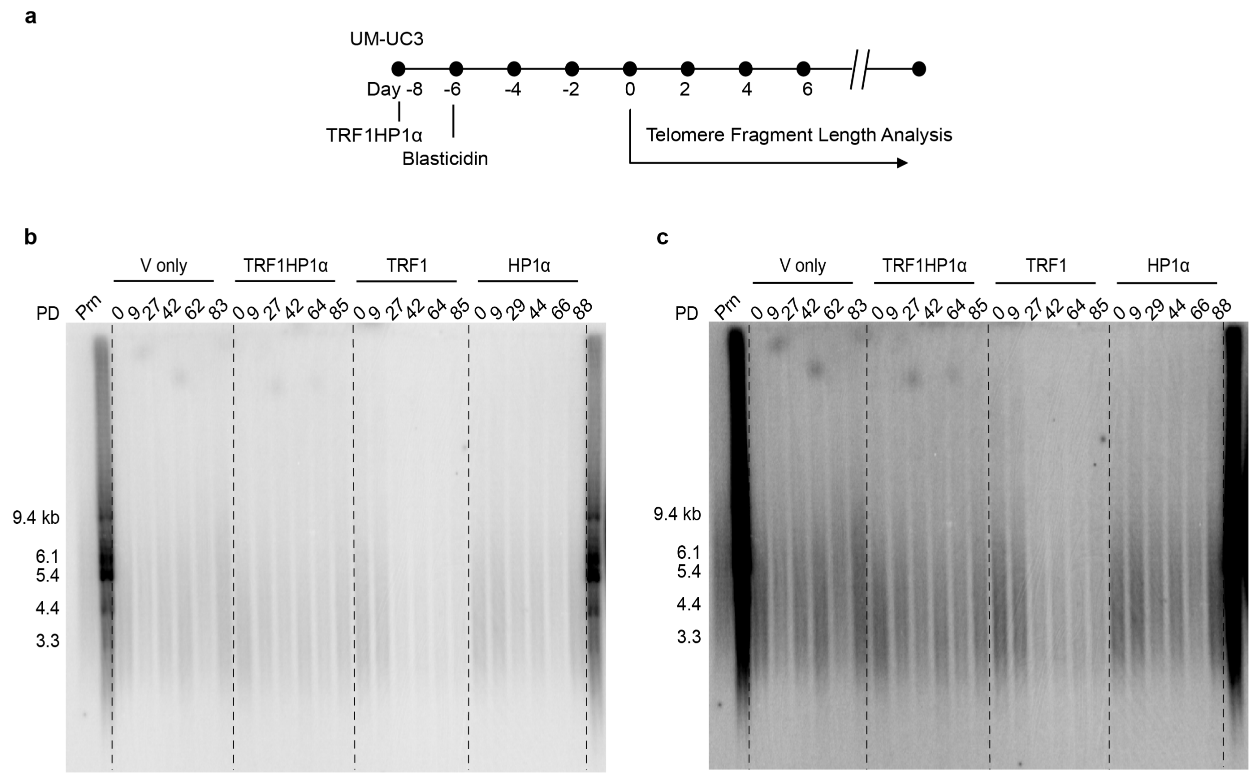

Supplementary Figure 3: Similar telomere length across multiple PDs. (a) Experimental set-up in UM-UC3 cells. **(b)** Telomere lengths distributions (Southern blotting). Vonly, TRF1HP1α, TRF1 or HP1α were without WT hTR overexpression; Prn: parental cell line. Similar findings were observed in two independent experiments. In the absence of additional WT hTR, because UM-UC 3 telomeres are quite short, it is possible to have compensation of HP1α heterochromatin at telomeres across multiple population doublings, and / or selective pressure against cells with shortened telomeres, resulting in similar telomere length over time. **(c)** Higher contrast exposure of **(b).**

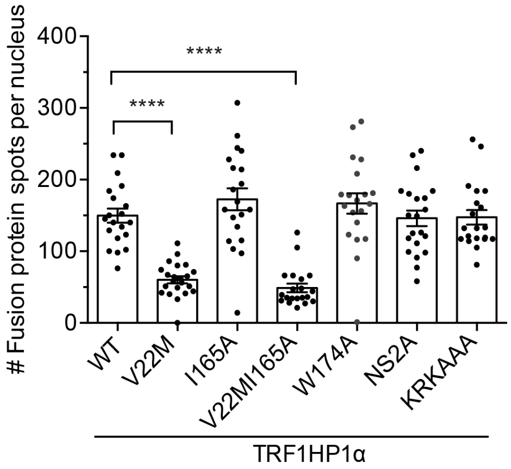

Supplementary Figure 4. Characterization of WT TRF1HP1α versus mutant TRF1HP1α at the telomeres. Quantification of number of fusion protein spots per nucleus. ****$p < 0.0001$, consistent with % area of fusion protein per nucleus shown in Fig. 3. Significance is assessed by one-way ANOVA and Dunnett's multiple comparison test with 95% confidence level. Error bars represent s.e.m. n = ~20 nuclei per group.

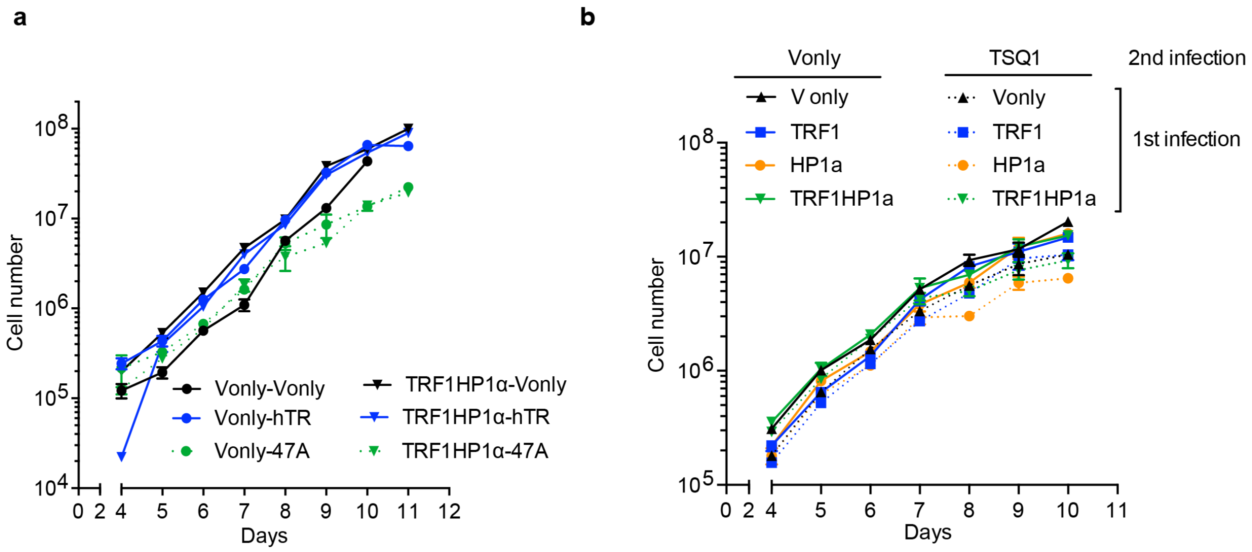

Supplementary Figure 5: Effects of mutant hTR on the growth of UM-UC3 cells. Growth curves show that treatment with **(a)** mutant hTR 47A (each data point presents average of 2-4 replicates) or **(b)** TSQ1 (each data point presents average of 6 replicates) results in minimal to only a mild growth defect in all groups. Error bars represent s.e.m.

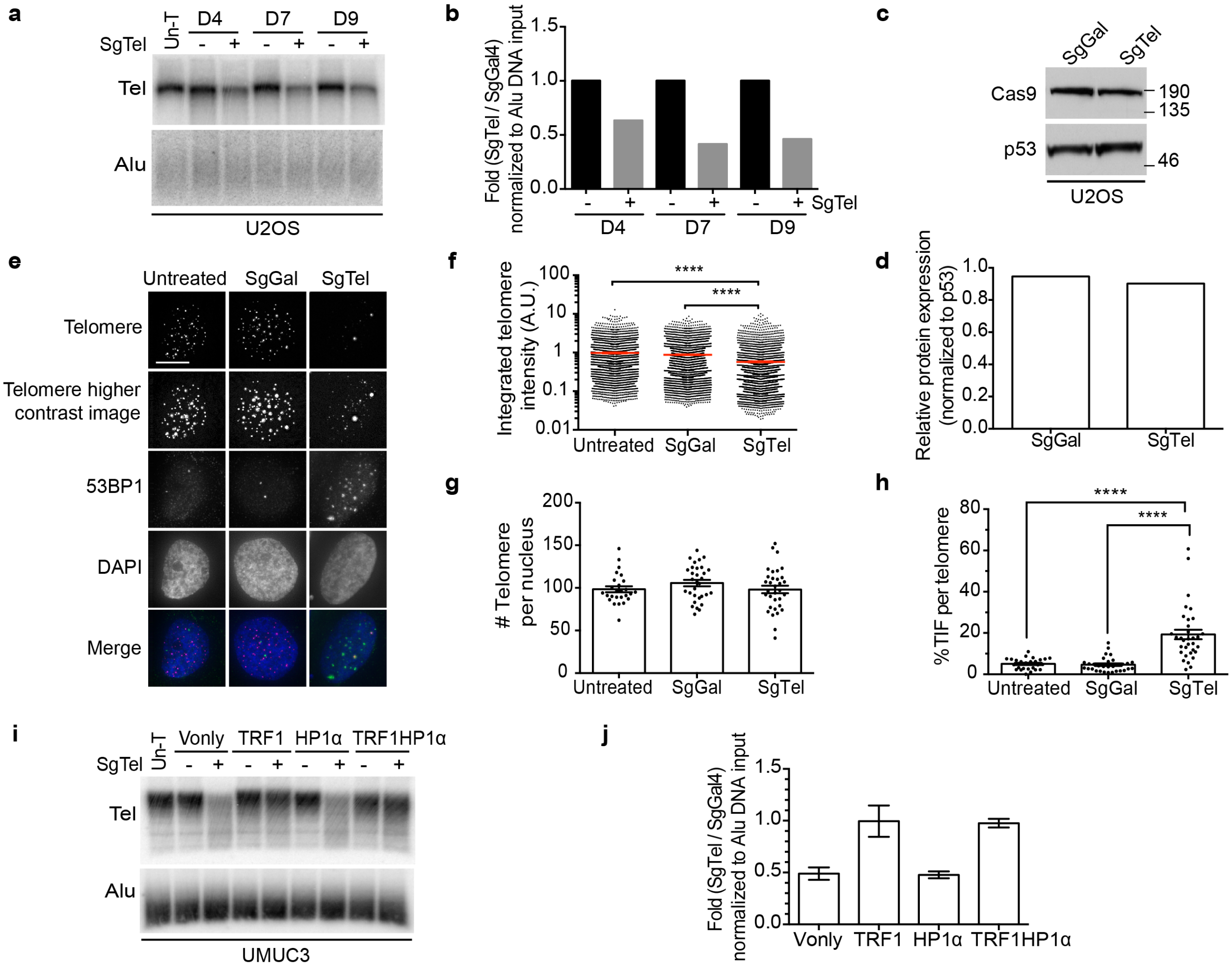

Supplementary Figure 6: TRF1 is sufficient to protect telomeres from CRISPR-induced telomere damage.

(a-h) Efficiency of CRISPR-induced telomeric DNA cutting in U2OS at Day 4, 7 and 9. **(a)** SgTel (+), SgGal4 negative control (-), Un-T (Untreated). Tel: telomeric probe; Alu intensity for input normalization; **(b)** Quantification. **(c-d)** Cas9 protein expression and quantification using p53 as loading control on Day 6. **(e)** TIF analysis (representative images) for untreated parental cells, SgGal or SgTel on Day 8. Bar: 10 μm. Telomere (TelC-Cy3; magenta in merged image); 53BP1 (green); DAPI (blue) **(f)** SgTel resulted in less integrated telomere intensity compared to the controls (untreated and SgGal) ****$p < 0.0001$ untreated (n = 2,577 telomeres); SgGal (n = 3,277 telomeres); SgGal (n = 3,239 telomeres). A.U. (arbitrary unit). Meanwhile, **(g)** numbers of telomere remain similar across groups: untreated (n = 26 nuclei); SgGal (n = 31 nuclei); SgTel (n = 33 nuclei). **(h)** SgTel resulted in increased TIF. ****$p < 0.0001$. Significance is assessed by one-way ANOVA and Dunnett's multiple comparison test with 95% confidence level. **(i-j)** In UM-UC3, TRF1 alone is sufficient to inhibit SgTel cutting on Day 5 (n = 3). Quantification shown in **(j)**. **(f-h, j)** Error bars represent s.e.m.

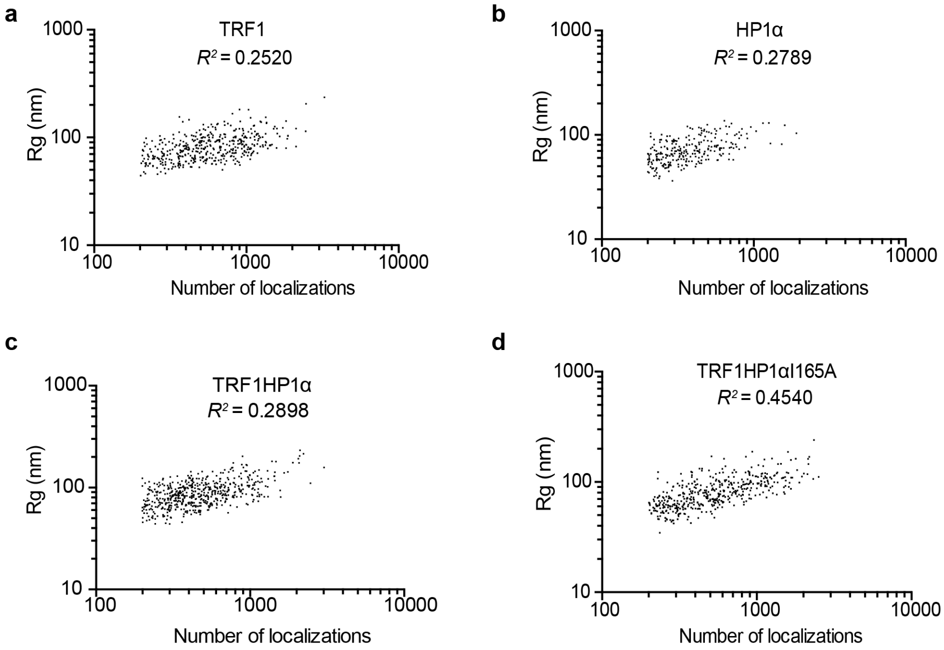

Supplementary Figure 7: Numbers of localization spots per Rg (nm).
Rg versus number of localization spots (dots on graph). Average localization spots: **(a)** TRF1 **(**664) with standard deviation (s.d.) 404 **(b)** HP1α (420) with s.d. 236 **(c)** TRF1HP1α (544) with s.d. 352 **(d)** TRF1HP1αI165A (639) with s.d. 428. R^2 corresponds to the linear regression of log transformed "number of localizations".

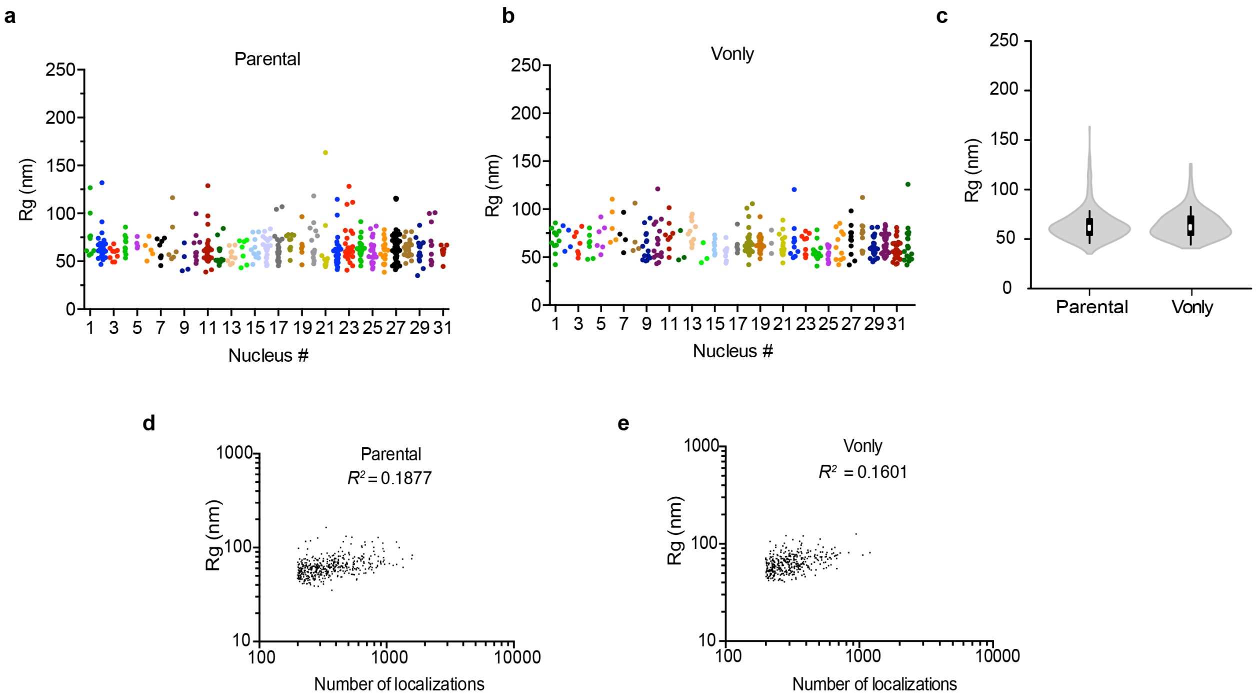

Supplementary Figure 8: Average Rg distributions are similar in parental and Vonly cells. **(a-b)** Rg of individual telomeres (dots) corresponding to **a)** Parental (*n* = 31 nuclei, 441 telomeres; average Rg 61.1 nm) or **(b)** Vonly (*n* = 32 nuclei, 352 telomeres; average Rg 64.5 nm). Y-axis: Rg (nm). X-axis: nucleus index. Each individual nucleus is distinguished by a different color. Each dot corresponds to one telomere. **(c)** Distribution of Rg (nm) in a violin plot shows frequency (width of density plot), median (white dot), interquartile range (bar) and 95% confidence interval (line). **(d-e)** Rg versus number of localization spots per individual telomere (dots on graph). Average localization spots: **(d)** Parental (400) with standard deviation (s.d.) 220 **(e)** Vonly (327) with s.d. 133. R^2 corresponds to the linear regression of log transformed “number of localizations”.

Fig. 5a - TRF2

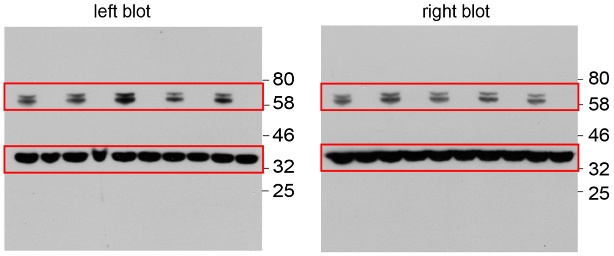

Supplememtary Fig. 2a

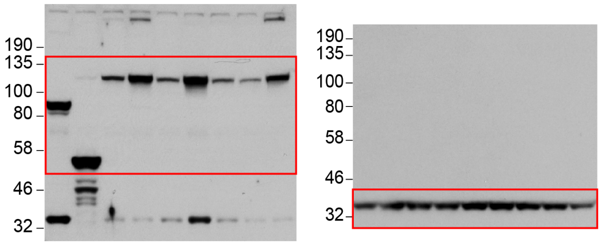

Supplememtary Fig. 2b

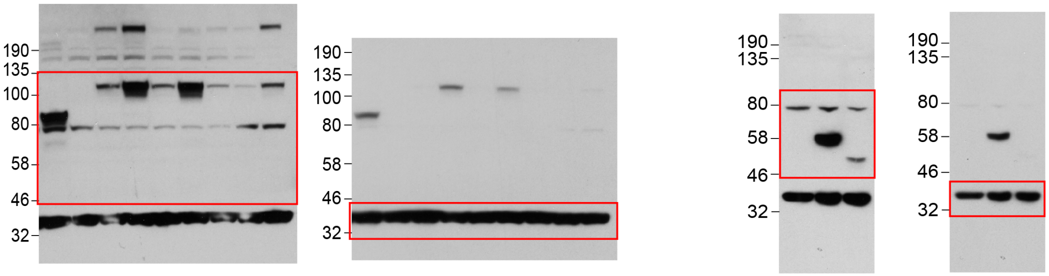

Supplememtary Fig. 2c

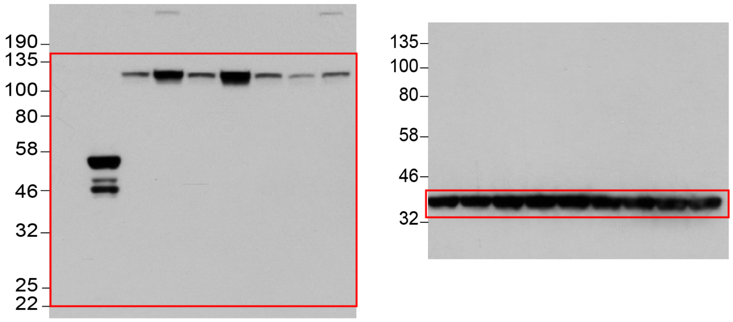

Supplememtary Fig. 6c

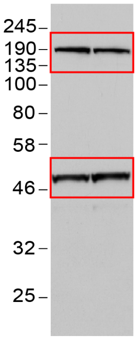

Supplementary Figure 9: Uncropped western blots.

Fig. 2b

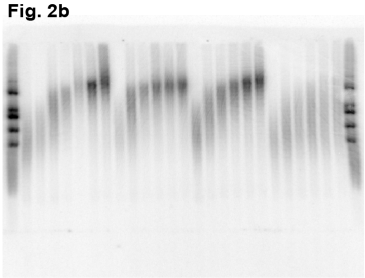

Fig. 3f

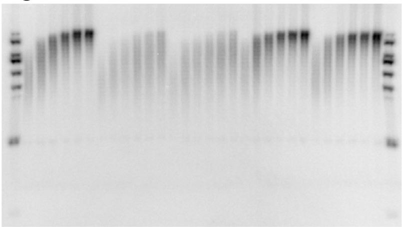

Fig. 3g

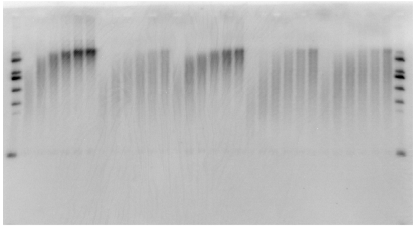

Fig. 6a

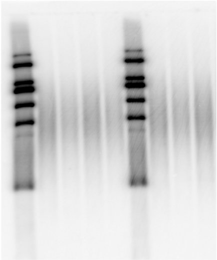

Supplementary Fig. 3b

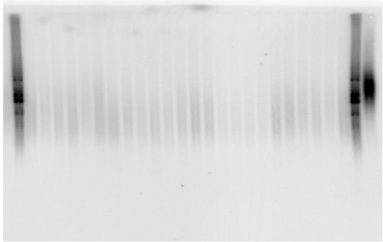

Supplementary Fig. 6a

Tel

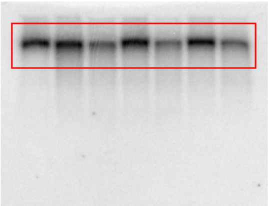

Alu

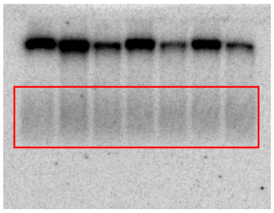

Supplementary Fig. 6i

Tel

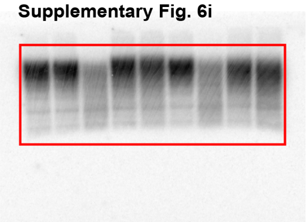

Alu

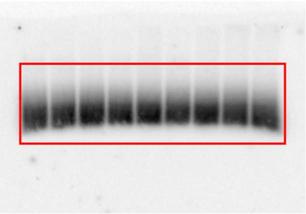

Supplementary Figure 10: Uncropped gels.
